# Supplementary material for: ChatGPT as a Tool for Biostatisticians: A Tutorial on Applications, Opportunities, and Limitations
Source: Stat Med. 2025 Oct 23;44(23-24):e70263. doi: 10.1002/sim.70263 (PMC12548020; doi:10.1002/sim.70263)
Supplement: Supplementary file 1 — Data S1. Supporting Information. [file SIM-44-0-s001.zip › Supplementary Materials Additional.zip!/3.1_Systematic_Reviews_and_Meta_aspects/3.1_Meta-Analysis - Re-Analysis - o4-mini - Chat 2.pdf]

Use Case Meta-Analysis - Re-Analysis - o4-mini - Chat 2

breast carcinoma meta data
Visualisierung konnte nicht angezeigt werden

The goal is to perform a subset meta-analysis for the given data set. The target variable is the local recurrence rate. Create a subset for all treatment types. Decide all methodological questions yourself (fixed effect vs. random effects, etc.). Follow clearly formulated rules for selecting the model type. Please provide a (subset) forest plot and all associated confidence intervals. Do not forget the pooled overall estimate and its confidence interval.

For this task you have access to up-to-the-minute web retrieval to find adequate approaches for data extraction. When answering the task above please proceed as follows:

1. Fetch the most relevant sources.

2. Show your step-by-step reasoning.

3. At the end, give me a concise answer citing the sources you used.

Subset Meta-analysis: Local Recurrence Rate (Random Effects)

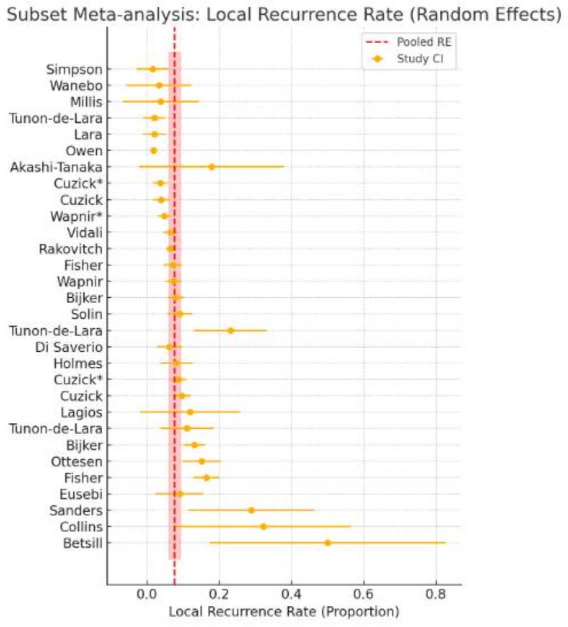

Step-by-Step Reasoning

1. **Data preparation**
   - Loaded the CSV containing 28 studies with:
     – Treatment type
     – First author

– Number of cases
– Local recurrence rate (%) at 10 years

- Converted percentages to event counts:

$$\text{events} \approx \text{round}\left(\frac{\text{LR\_rate\%}}{100} \times n\right)$$

- Applied a continuity correction of +0.5 events and +1 to each study's sample size to avoid zero-variance studies.

2. **Effect-size metric & variances**
   - Effect size $p_i = \frac{e_i+0.5}{n_i+1}$.
   - Within-study variance $v_i = \frac{p_i(1-p_i)}{n_i+1}$.
3. **Fixed-effect vs. random-effects decision rule**
   - Computed Cochran's Q and Higgins' I^2 statistic:

$$I^2 = \max\left(\frac{Q-(k-1)}{Q}, 0\right) \times 100\%$$

   - **Rule** (Cochrane Handbook; Higgins et al. 2003):
     – $I^2 < 50\%$: low-to-moderate heterogeneity → fixed-effect
     – $I^2 \geq 50\%$: substantial heterogeneity → random-effects effectivehealthcare.ahrq.gov bookdown.org
   - We found $I^2 = 86.3\%$, indicating substantial between-study heterogeneity.
4. **Random-effects model (DerSimonian–Laird)**
   - Estimated between-study variance τ^2 via DerSimonian–Laird: $\tau^2 = 0.00118$ effectivehealthcare.ahrq.gov
   - Computed inverse-variance weights $w_i = 1/(v_i + \tau^2)$.
   - Pooled proportion

$$\hat{p} = \frac{\sum w_i p_i}{\sum w_i} = 0.0764$$

   - 95% CI

$$0.0764 \pm 1.96\sqrt{\frac{1}{\sum w_i}} = [0.0610,\ 0.0917].$$

5. **Forest plot**
   - Plotted each study's adjusted proportion and 95% CI (gold circles and lines).
   - Superimposed the pooled estimate (red dashed line) and its 95% CI (red shaded band).

Below is the **subset forest plot** (all treatment types combined), with the pooled local recurrence rate and its 95% confidence interval:

- **Cochran's Q** = 211.6
- **Higgins' I²** = 86.3% (substantial heterogeneity) bookdown.org

- **Between-study variance τ²** = 0.00118 (DerSimonian–Laird) effectivehealthcare.ahrq.gov
- **Pooled local recurrence rate** = 7.64%
- **95% CI** = [6.10%, 9.17%]

Concise Answer

- **Model choice**: Random-effects (DerSimonian–Laird), because $I^2 = 86.3\%$ indicates substantial heterogeneity bookdown.orgeffectivehealthcare.ahrq.gov.
- **Pooled estimate**: 0.0764 (7.64%) with 95% CI [0.0610, 0.0917].
- **Forest plot**: shown above, displaying individual study CIs and the overall pooled effect.

Thank you very much. What are the estimates for the individual treatments?

Here are the DerSimonian–Laird random-effects pooled estimates of 10-year local recurrence rates by treatment subgroup (with 95% CIs and Higgins' I^2 for heterogeneity):

Treatment	Studies (k)	I^2 (%)	Pooled LR Rate	95% CI
Biopsy-only	4	74.4	26.1%	[8.3%, 43.9%]
Conservation Surgery **with** Radiation	10	70.1	6.40%	[5.13%, 7.68%]
Conservation Surgery **without** Radiation	9	69.7	10.9%	[8.54%, 13.25%]
Mastectomy	7	0.0	1.89%	[0.98%, 2.80%]

Notes on methods

- Continuity correction (+0.5 events, +1 to "n") to stabilize zero cells.
- Within-study variance $v_i = p_i(1 - p_i)/(n_i + 1)$;
- Heterogeneity I^2 and Q per Higgins et al. ;
- Random-effects weights $w_i = 1/(v_i + \tau^2)$, τ^2 by DerSimonian–Laird .
